# Enhanced Microwave-Absorbing Property of Honeycomb Sandwich Structure with a Significant Interface Effect

**DOI:** 10.3390/ma15165741

**Published:** 2022-08-19

**Authors:** Yiming Zhao, Yimeng Shan, Guoliang Ji, Yungang Sun, Weibin Shi, Minghang Li

**Affiliations:** 1 State-Owned Wuhu Machinery Factory, Wuhu 241000, China; 2 Science and Technology on Thermostructural Composite Materials Laboratory, Northwestern Polytechnical University, Xi’an 710072, China

**Keywords:** microwave absorption, honeycomb sandwich structure, interface effect

## Abstract

Honeycomb sandwich structures (HSSs) are excellent candidates for light and efficient microwave-absorbing materials. In this work, we design an HSS using SiO_2_ fiber-reinforced epoxy resin (SiO_2f_/ER) composites as both the top and bottom layers to improve the impedance matching with free space. Target dielectric properties of the honeycomb and coated lossy material of the HSS were calculated based on the multilayer transmission line theory, metal backplane model, and homogenization theory. In addition, the interface effect between the SiO_2f_/ER and honeycomb of the HSS was discussed theoretically, experimentally, and numerically, indicating a 1–4% contribution of microwave absorption resulting from the interface. By analyzing the equivalent resistance, equivalent capacitance, as well as equivalent inductance, the enhanced microwave absorption of HSS is attributed to the formation of the interfacial transition zone, which benefits both impedance matching and electromagnetic loss.

## 1. Introduction

The offensive and defensive battles of radar and stealth aircraft are constantly being staged on the modern information battlefield, originating from the second world war [1,2]. The first generation of radar-absorbing materials, represented by ferrite-based coatings, was manufactured by the Germans in response to the early radar devices successfully developed by the Allies. Therefore, the rapid development of radar detection technology promoted the design of a new generation of absorbing materials/structures [3,4,5,6].

Aramid honeycomb sandwich structure (AHSS) has relatively low dielectric constant and dielectric loss, and thus exhibits excellent microwave transmission properties when quartz fiber-reinforced resin is used as top and bottom panels to ensure the detection range and strike accuracy of the radar [7]. AHSS also exhibits excellent microwave absorption properties when conductive coatings such as carbon nanotube, carbon black, conductive polymers, or magnetic powders are attached to the inner wall of the honeycomb to reduce the radar cross section (RCS) [8,9,10,11,12]. At the same time, the high flexural strength/mass ratio and strong anti-instability ability of AHSS determine that it plays an important structural and functional integration role of “wave-transmitting spear and stealth shield” on stealth aircraft [13,14,15]. In addition to the mesoscopic scale design of AHSS, the microstructure design of conductive fillers can also serve as an important means to improve electromagnetic absorption and shielding capabilities [16,17].

According to the literature, many researchers have explored the effect of the honeycomb structure size, thickness, and electrical properties of coated lossy material, as well as panel matching layer thickness, on microwave absorption efficiency thanks to the excellent design-ability of the honeycomb sandwich structure [18,19]. At the same time, a frequency selective surface is designed and introduced into AHSS to achieve broadband tunable microwave absorbing properties [20]. The interface between the panel matching layer and the honeycomb produces an interface polarization effect in the alternating electromagnetic field, which is known as the Maxwell–Wagner–Sillars (MWS) effect, and can also consume microwave energy [21,22,23,24]. However, the MWS effect between the panel matching layer and honeycomb has not yet been discussed and quantified.

In this paper, the honeycomb sandwich structure with SiO_2f_/ER as matching panels based on the design principle of multilayer transmission line theory and the metal backplane model is proposed theoretically, experimentally, and numerically to quantify the role of the interface effect. The respective impedance, resistance, capacitance, and inductance, both when considering the interface effect and not considering the interface effect, are calculated and used to further analyze the underlying mechanism of electromagnetic loss at the interface.

## 2. Experimental and Simulation Method

### 2.1. Material Fabrication

**Figure 1:**
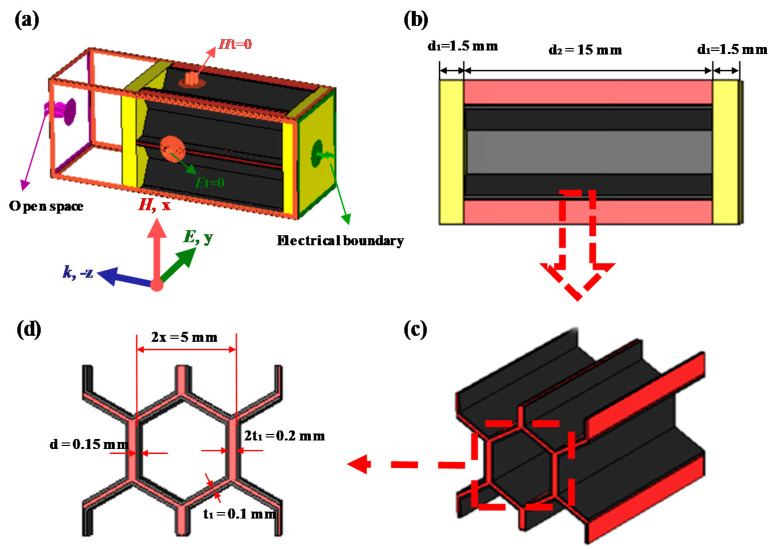
Schematic of a unit cell in the designed honeycomb sandwich structure: (**a**) 3D view, (**b**) side view, (**c**) honeycomb core, and (**d**) geometry of the honeycomb core.

Figure 1 shows the schematic of a unit cell in the designed honeycomb sandwich structure. Firstly, the SiO_2f_/ER layer with a thickness d_1_ of 1.5 mm serving as the panel matching layer was prepared by the vacuum pressure impregnation process, in which the mass fraction of epoxy resin is 35% and the corresponding dielectric constant *ε* = 3.4 (1-j0.025) at 10 GHz. Secondly, the conductive coating with a thickness d of 0.15 mm was prepared using a high-speed mixer. Here, the mass fraction of conductive carbon black powders (Nanjing Xianfeng Chemical Co., Ltd., Nanjing, China) is 28% and the corresponding mass fraction of epoxy resin is 72%. Thirdly, a conductive coating was introduced into inner wall of honeycomb via the dip-coating process; here, the dipping time is 1 min, following by drying in a blast drying oven at 90 °C for 10 min. By repeating the dip-coating process on the opposite face to the previous time, the uniformity of the coating thickness along the honeycomb height direction is ensured and the coating thickness can be calculated based on the honeycomb weight gain. Fourthly, the panel and honeycomb are bonded by adhesive film, whose thickness is 0.2 mm and with the corresponding dielectric constant *ε* = 3.5 (1-j0.02) at 10 GHz, which can be studied together with the panel.

### 2.2. Characterization, Simulation, and Calculation

The respective dielectric properties of the conductive coating and the corresponding honeycomb core were determined by a vector network analyzer (VNA, MS4644A; Anritsu, Atsugi, Japan) in X-band (8.2–12.4 GHz), using a 22.86 mm × 10.16 mm rectangular waveguide. The reflection loss experimental results in X-band of the fabricated honeycomb sandwich structure with dimensions of 180 mm × 180 mm × 18 mm were measured using the free space method. CST Microwave Studio was used to perform numerical simulation on the honeycomb sandwich structure based on the finite integration technique.

Three-layer transmission line theory and the metal backplane model were used to calculate the reflection loss (*RL*), impedance, and corresponding microwave absorption efficiency (*A*(ω)) of the honeycomb sandwich structure, which can be expressed as follows [25,26]:

(1) $$RL=20\log_{10}\left|\frac{Z_{3}-Z_{0}}{Z_{3}+Z_{0}}\right|=10\log_{10}\left(1-A\left(\omega \right)\right)$$

(2) $$Z_{1}=\sqrt{1/\varepsilon_{1}}\tanh\left[j2\pi fd_{1}\sqrt{\varepsilon_{1}}/c\right]$$

(3) $$Z_{2}=\sqrt{1/\varepsilon_{2}}\frac{Z_{1}+\sqrt{1/\varepsilon_{2}}\tanh\left[j2\pi fd_{2}\sqrt{\varepsilon_{2}}/c\right]}{\sqrt{1/\varepsilon_{2}}+Z_{1}\tanh\left[j2\pi fd_{2}\sqrt{\varepsilon_{2}}/c\right]}$$

(4) $$Z_{3}=\sqrt{1/\varepsilon_{3}}\frac{Z_{2}+\sqrt{1/\varepsilon_{3}}\tanh\left[j2\pi fd_{3}\sqrt{\varepsilon_{3}}/c\right]}{\sqrt{1/\varepsilon_{3}}+Z_{2}\tanh\left[j2\pi fd_{3}\sqrt{\varepsilon_{3}}/c\right]}$$

where *Z*_i_ is the normalized impedance (the ratio of complex impedance to vacuum impedance) of layer i from the bottom up; *ε*_i_ and *d*_i_ are the relative complex permittivity (the ratio of complex permittivity to vacuum permittivity) and thickness of layer i, respectively; *f* is the frequency of microwave; *c* represents the speed of light in the free space; and ω is the angular frequency of microwave.

## 3. Results and Discussion

### 3.1. Target Dielectric Properties of the Honeycomb and Coated Lossy Material

**Figure 2:**
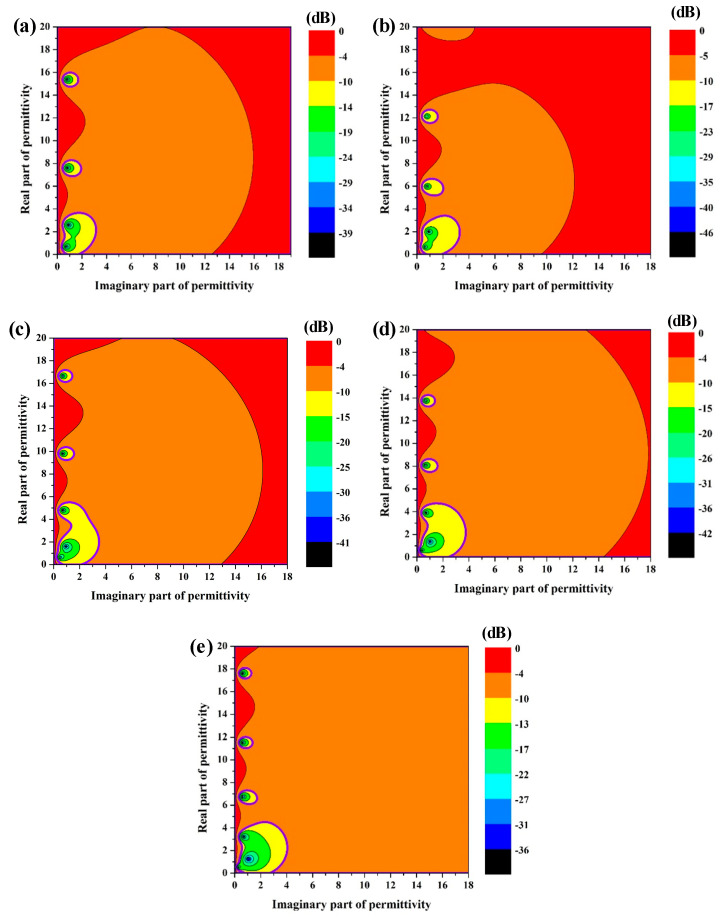
Reflection loss spectrums of a honeycomb core 15 mm in height with different equivalent dielectric properties: (**a**) 8.2 GHz, (**b**) 9.2 GHz, (**c**) 10.2 GHz, (**d**) 11.2 GHz, and (**e**) 12.2 GHz.

For the three-layer absorbing structure in this work, the target equivalent dielectric properties of the honeycomb core 15 mm in height can be calculated according to Equations (1)–(4) when the thickness of the top and bottom panels is 1.5 mm and the corresponding dielectric constant *ε* = 3.4 (1-j0.025) at 10 GHz. Figure 2 exhibits the contour plot of *RL* corresponding to different dielectric properties of the honeycomb core at 8.2 GHz, 9.2 GHz, 10.2 GHz, 11.2 GHz, and 12.2 GHz, respectively. The bold purple circle in Figure 2 shows the range where *RL* is less than −10 dB. It can be concluded that lower equivalent dielectric properties of the honeycomb can significantly increase the target range of equivalent dielectric properties.

Based on the strong interference theory and long-wave approximation, the equivalent dielectric property of a honeycomb *ε*_z_ 15 mm in height can be expressed as follows [27]:

(5) $$\varepsilon_{z}=\frac{t_{1}}{x}\left(2-\frac{d}{x-t_{1}}\right)\varepsilon_{2}+{\left(1-\frac{t_{1}}{x}\right)}^{2}\varepsilon_{p}$$

(6) $$\varepsilon_{p}=\frac{d}{x-t_{1}}\left(2-\frac{d}{x-t_{1}}\right)\varepsilon_{1}+{\left(1-\frac{d}{x-t_{1}}\right)}^{2}$$

where *ε*_1_ and *ε*_2_ are the relative complex permittivity of coated lossy material and the aramid fiber, respectively, where *ε*_2_ = 2 (1-j0.015) at 10 GHz; *ε*_z_ and *ε*_p_ are the equivalent relative complex permittivity of the honeycomb core and coated lossy material combined with its surrounding air column, respectively; *t*_1_ is the thickness of the aramid honeycomb core wall; and *x* represents half the distance between the opposite wall of the aramid honeycomb core.

**Figure 3:**
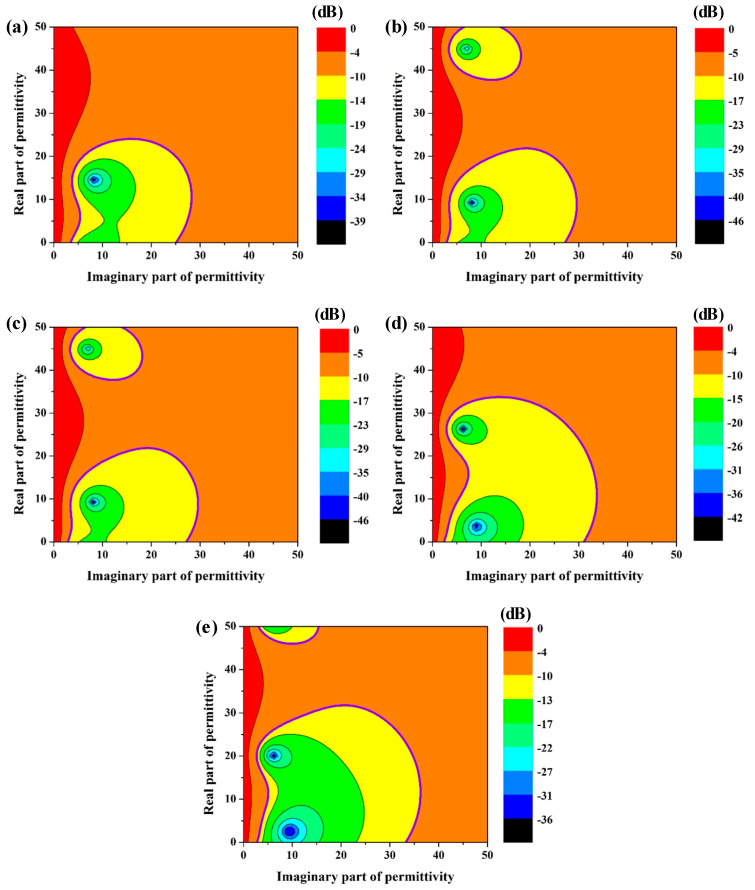
Reflection loss spectrums of the coated lossy material with different dielectric properties: (**a**) 8.2 GHz, (**b**) 9.2 GHz, (**c**) 10.2 GHz, (**d**) 11.2 GHz, and (**e**) 12.2 GHz.

Therefore, according to Equations (5) and (6), the target dielectric range of coated lossy material in Figure 3 can be obtained from the target equivalent dielectric range of the honeycomb core, which is shown in Figure 2. As shown in Figure 3, the existence of the high-porosity aramid honeycomb framework significantly increases the target dielectric range of the coated lossy material, which implies a more easily tunable microwave-absorbing performance.

### 3.2. Microwave Absorption of the Honeycomb Sandwich Structure

**Figure 4:**
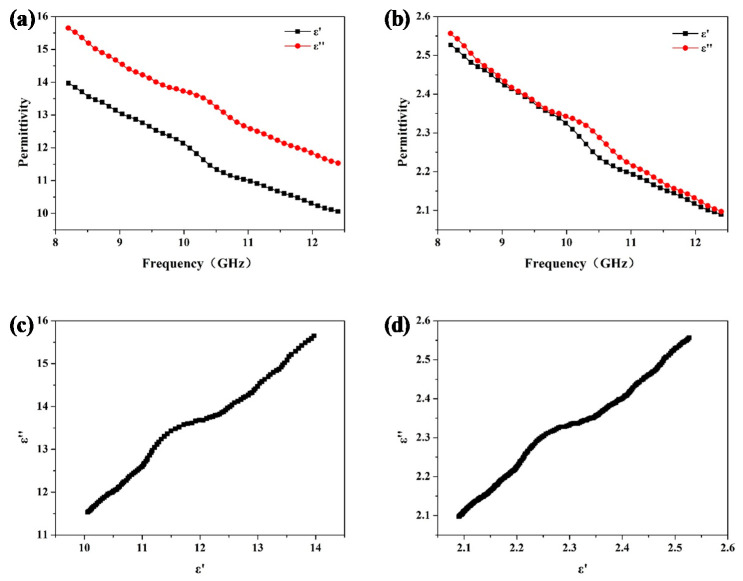
Dielectric properties (**a**,**b**) and cole–cole diagram (**c**,**d**) of the coated lossy material and corresponding honeycomb.

In order to improve microwave absorption property of the honeycomb sandwich structure, a carbon black/ER composite with dielectric properties in the target range of coated lossy material obtained from Section 3.1 is introduced into the honeycomb inner wall with a coating thickness of 0.15 mm. The respective dielectric properties of the coated lossy material and corresponding honeycomb are shown in Figure 4a,b. It is obvious that the coated lossy material and corresponding honeycomb have a significant frequency dispersion effect, thus exhibiting a greater hysteresis effect of polarized charges and enhancing the loss of microwave energy. For dielectric lossy material in this work, the cole–cole diagram reflects its microwave loss mechanism. Specifically, one semicircle indicates one Debye polarization and relaxation process and one straight line indicates a conductive loss dominated loss mechanism. Figure 4c,d exhibits the respective cole–cole diagrams of the coated lossy material and corresponding honeycomb, which indicate a loss mechanism dominated by conductive loss.

**Figure 5:**
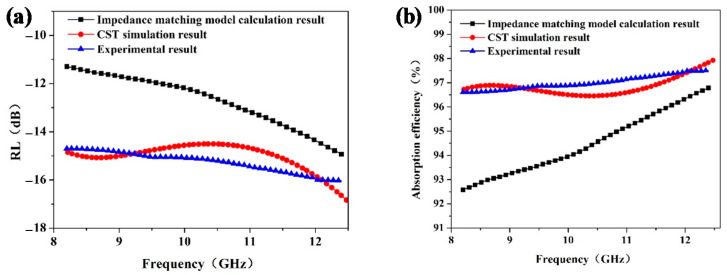
Reflection loss (**a**) and corresponding microwave absorption efficiency (**b**) of the impedance matching model calculation result, CST simulation result, and experiment result.

Figure 5 exhibits the reflection loss and corresponding absorption efficiency of the honeycomb sandwich structure obtained from the impedance matching model according to Equations (1)–(4), as well as the CST simulation method and experiment. The CST simulation result and experimental result take into account the effect of the interface between the SiO_2_/ER panel and honeycomb in the sandwich structure. *RL* has a deviation of less than 1 dB in the X-band and the corresponding deviation of microwave absorption efficiency is less than 1%, verifying the accuracy of the simulation results. Interestingly, the impedance matching model calculation result when the interface effect between the SiO_2_/ER panel and honeycomb is not considered exhibits a relatively low reflection loss and poor microwave absorption efficiency. The presence of the interface between the panel and honeycomb reduces the *RL* by 1–4 dB in the X-band and the corresponding microwave absorption efficiency increased by 1%–4%, which implies that the interface plays an important role in the absorption of microwave energy. Next, we will further investigate the role of the interface effect from impedance, resistance, capacitance, and inductance with and without the interface effect.

### 3.3. Role of the Interface Effect

**Figure 6:**
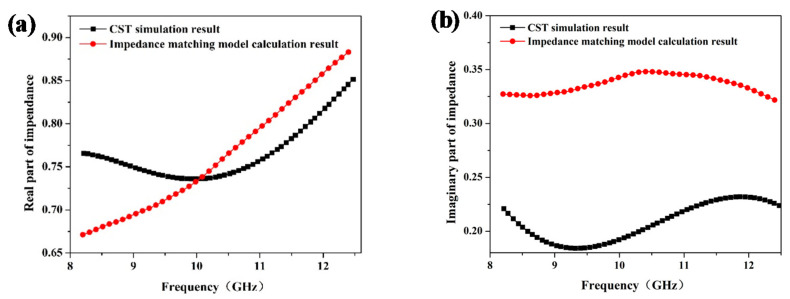
Real (**a**) and imaginary (**b**) part of impedance obtained from the CST simulation result and impedance matching model result.

To further investigate the role of the interface effect in the honeycomb sandwich structure, respective normalized impedance of the CST simulation result and impedance matching model calculation result are exhibited in Figure 6. The real part of impedance represents the ability to dissipate energy from microwave and the imaginary part of impedance represents the ability to store microwave energy. When considering the interface effect, the CST simulation result exhibits a higher overall real part of impedance as well as a relatively lower imaginary part of impedance, which indicate excellent impedance matching performance. Interestingly, at the frequency point of 10 GHz, the interface has almost no effect on the real part of impedance, while it significantly reduces the imaginary part of impedance. This interesting phenomenon indicates that the presence of the interface reduces the ability to store charge and the interface acts as a charge transporter between the honeycomb and panel.

**Figure 7:**
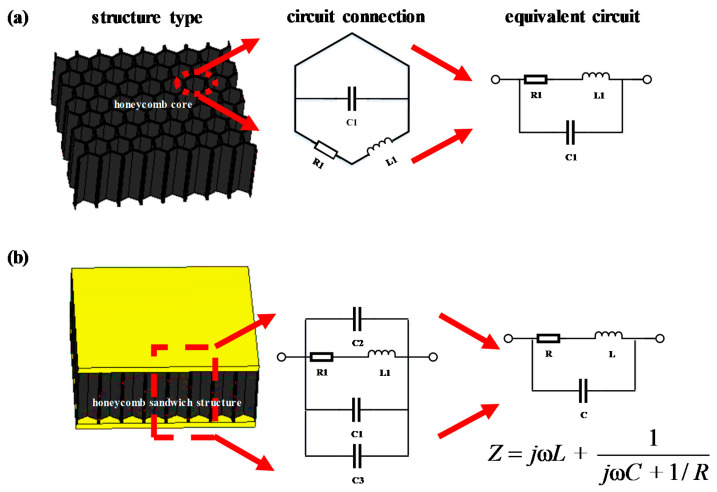
Equivalent circuit model for the (**a**) honeycomb and (**b**) honeycomb sandwich structure.

According to the structural characteristics of the honeycomb core and honeycomb sandwich structure, the respective equivalent circuit models are shown in Figure 7. Here, C1 represents the capacitance between opposing honeycomb walls; R1 and L1 represent the resistance and inductance, respectively; and C2 and C3 represent the capacitance of the upper and lower panel, respectively. According to the equivalent circuit, the impedance of the honeycomb sandwich structure can be expressed as follows:

(7) $$Z=j\omega L+\frac{1}{j\omega C+1/R}$$

According to Equation (7), the respective resistance, capacitance, and inductance can be obtained from the real and imaginary part of impedance represented in Figure 6. Specifically, the resistance, capacitance, and inductance at 10 GHz can be calculated from the values of the real and imaginary part of impedance at 9.99 GHz and 10.01 GHz when the changes in resistance, capacitance, and inductance caused by 0.02 GHz are considered negligible.

**Figure 8:**
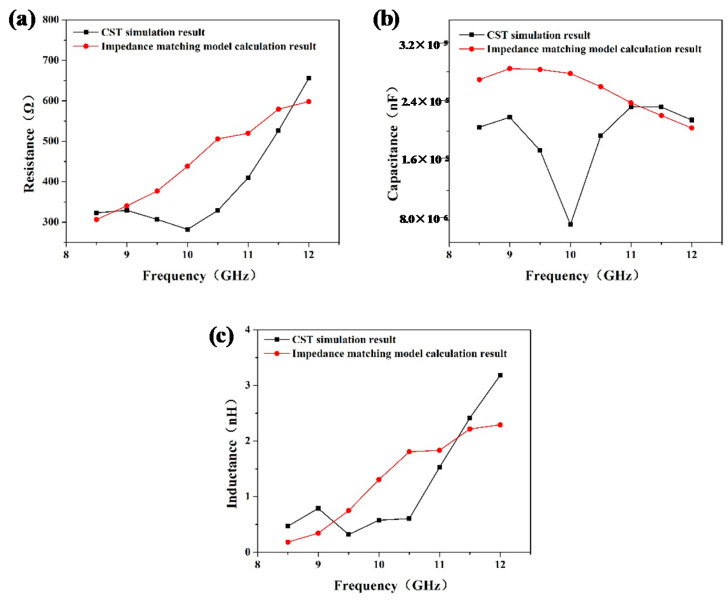
Resistance (**a**), capacitance (**b**), and inductance (**c**) obtained from the CST simulation and impedance matching model.

The corresponding calculation results at different frequency points in the X-band are shown in Figure 8. Obviously, the overall resistance, capacitance, and inductance decrease when considering the interface effect. The charge accumulated at the interface can easily migrate on both sides of the interface. Interestingly, the resistance, capacitance, and inductance show minimal values near 10 GHz, which means a more significant interface effect.

**Figure 9:**
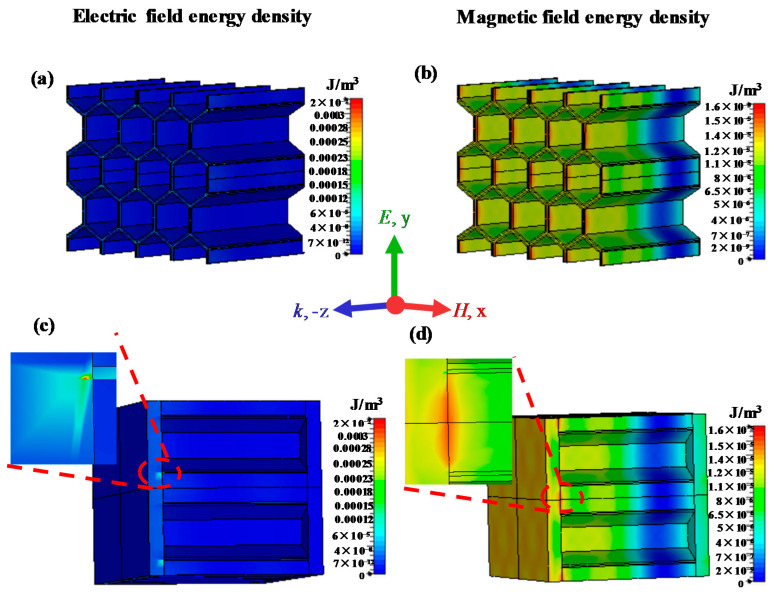
Simulated distributions of electric field energy density (left) and magnetic field energy density (right) of microwave-absorbing structures: (**a**,**b**) honeycomb core; (**c**,**d**) honeycomb sandwich structure.

Figure 9 demonstrates the distribution of the electric field energy density and magnetic field energy density at 10 GHz, which are analyzed to investigate the underlying physical mechanism of microwave absorption considering the interface effect. Here, the incident electromagnetic field is a uniform plane wave and the corresponding incident electric field energy density is 2 × 10^−2^ J/m^3^. Compared with the microwave absorption without considering the interface effect, a strong coupling of the electric field and magnetic field emerges at the interface area of the panel and honeycomb. In particular, the energy concentration of electric and magnetic field occurs in both sides of the interface thin layer region. Thus, the existence of the interface transition zone, on one hand, improves the impedance matching and enables more microwave incident into the material. On the other hand, the increases in energy density of the electric field and magnetic field enhance the electromagnetic coupling effect and increase the loss of microwave energy. Therefore, the interface play an important role in the enhancement of microwave absorption efficiency in the honeycomb sandwich structure.

**Figure 10:**
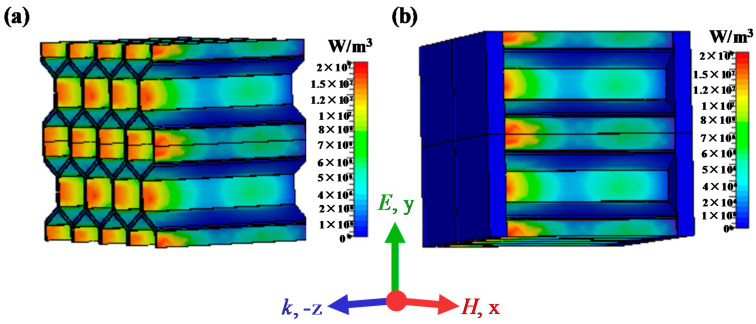
Simulated distributions of the power loss density of the (**a**) honeycomb core and (**b**) honeycomb sandwich structure.

Furthermore, the power loss density distributions of the honeycomb sandwich structure and the corresponding honeycomb inside the sandwich structure are shown in Figure 10. The interface between the upper panel and honeycomb exhibits a strong electromagnetic loss, which is attributed to the strong electromagnetic coupling effect. Therefore, the existence of the interface transition zone reduces the overall resistance, capacitance, and inductance, which optimize impedance matching and enable more microwave incident into the material. At the same time, an enhanced electromagnetic coupling effect occurs in the interface transition zone, which improves the energy loss of microwave.

## 4. Conclusions

A honeycomb sandwich structure based on multilayer transmission line theory and the metal backplane model with enhanced microwave absorption is proposed in this paper. Theoretical, experimental, and numerical results indicate that the interface between the panel and honeycomb play a vital role in the loss of microwave energy, where the existence of the interface contributes 1–4% of microwave absorption efficiency in the X-band. On one hand, the existence of the interface significantly reduces the resistance, capacitance, and inductance and more microwave can incident into material. On the other hand, enhanced incident microwave exhibits a strong electromagnetic coupling phenomenon in the thin layer region near the interface and enhances the loss of microwave energy. Therefore, interface design can be used as a supplementary means to further optimize the microwave absorption efficiency of the honeycomb sandwich structure.

## Data Availability

All the data are available within the manuscript.
